# *ACE* I/D and *MMP-7* A-181G variants and the risk of end stage renal disease

**Published:** 2017-03

**Authors:** Zohreh Rahimi, Hamed Abdi, Maryam Tanhapoor, Ziba Rahimi, Asad Vaisi-Raygani, Hamid Nomani

**Affiliations:** 1 Medical Biology Research Center, Kermanshah University of Medical Sciences, Kermanshah, Iran; 2 Department of Clinical Biochemistry, Medical School, Kermanshah University of Medical Sciences, Kermanshah, Iran

**Keywords:** *ACE*, *MMP-7*, Polymorphism, ESRD, Hypertension

## Abstract

The variants of angiotensin converting enzyme (*ACE*) and matrix metalloproteinases (MMPs) genes might be involved in the pathogenesis of end stage renal disease (ESRD) and hypertension. We studied the *ACE* insertion/deletion (I/D) and *MMP-7* A-181G variants in 99 unrelated ESRD patients and 117 individuals without renal complications from Western Iran with Kurdish ethnic background. The frequency of *ACE* I/D variants was not significantly different between ESRD patients and controls. However, the presence of *ACE* D allele increased the risk of hypertension in ESRD patients by 2.14-fold (P=0.036). The *MMP-7* -181 AG genotype increased the risk of ESRD by 2.04 times (P=0.026). The present study indicated the absence of an association between the *ACE* I/D polymorphism with the risk of ESRD. However, the ACE D allele increased the risk of hypertension in ESRD patients. Also, the present study suggests a role for *MMP-7* AG genotype in the pathogenesis of ESRD.

## INTRODUCTION

End stage renal disease (ESRD) is defined as an advanced chronic renal failure with declining renal function to approximately 10% of normal before initiation of dialysis or transplantation. There is a tight link between renal function with blood pressure and hypertension [1]. The renin angiotensin aldosterone system (RAAS) has a vital role in regulating blood pressure, electrolyte and fluid homeostasis [2]. The most studied polymorphism of the angiotensin converting enzyme (*ACE*) is an insertion/deletion polymorphism (*ACE* I/D) (rs1799752) that is associated with the highest systemic and renal ACE activity in the presence of D allele [3, 4]. The overall prevalence of the *ACE* D allele among Iranian populations has been reported to be 0.5886 [5]. The role of *ACE* I/D polymorphism in susceptibility to ESRD has been investigated in some studies but with inconsistency [1, 6-9]. Matrix metalloproteinases (MMPs) are a family of structurally related, zinc-dependent enzymes that play a crucial role in restructuring the extracellular matrix [10]. MMPs genes especially *MMP-7* and *MMP-20* mediate kidney aging and decreasing glomerular filtration rate [11]. The MMP-7 A-181G (rs11568818) polymorphism in the promoter region of *MMP-7* gene through affecting the binding of nuclear protein(s) modulates the transcription of the gene [10]. According to the literature there is no available study to investigate the role of *MMP-7* A-181G polymorphism in susceptibility to ESRD. We investigated association between *ACE* I/D and *MMP-7* A-181G variants with the risk of ESRD in a population from Western Iran with Kurdish ethnic background.

## MATERIALS AND METHODS

Sample consisted of 99 unrelated ESRD patients (mean age of 58.1 ± 13.3 years; 65 males, 34 females) and 117 individuals (mean age of 55.7 ± 7.3 years; 71 males, 46 female) without renal complications. The study was approved by the Ethics Committee of Kermanshah University of Medical Sciences and was in accordance with the principles of the Declaration of Helsinki II. 

Genomic DNA was extracted from peripheral blood leukocytes using the phenol-chloroform method. Genotyping of *ACE* I/D polymorphism was performed using polymerase chain reaction (PCR) as previously described [12]. The variants of *MMP-7* A-181G were detected using PCR-restriction fragment length polymorphism by EcoR I restriction enzyme [10]. 

The SPSS (SPSS Inc., Chicago, IL, USA) statistical software package version 16.0 was used for the statistical analysis. Statistical significance was assumed at the p<0.05 level.

## RESULTS AND DISCUSSION

Distribution of *ACE* I/D and *MMP-7* A-181G genotypes and alleles are depicted in Table 1. The frequencies of ACE genotypes and alleles were not statistically different comparing ESRD patients and controls. Some studies including a recent meta-analysis [1, 6-8] suggested an association between *ACE* I/D polymorphism and the risk of ESRD among both Asians and Caucasians. However, in a study with large sample among Caucasians with ESRD the *ACE* I/D polymorphism was not associated with progression of renal disease and ESRD [9]. The inconsistent reports among various ethnic groups might be due to the presence of genetic and environmental heterogeneity.

The frequency of *ACE* I/D genotypes and alleles in ESRD patients according to the history of hypertension (defined as blood pressure of at least 140/90 mmHg) indicated a significantly higher frequency of *ACE* DD genotype in ESRD patients with a history hypertension (n=17/29, 58.7%) compared to that in patients with normal blood pressure (n=27/66, 40.9%, P=0.035). Also, a significantly higher frequency of *ACE* D allele (n=45/58, 77.6%) was observed in ESRD patients with a history of hypertension than that in patients without a history of hypertension (n=81/132, 61.4%, P=0.034). The presence of ACE D allele increased the risk of hypertension in ESRD patients [OR=2.14, 95% CI 1.05- 4.35, P=0.036]. The risk of progression of chronic renal failure in the presence of high blood pressure has been demonstrated using clinical studies [13]. Also, in the presence of *ACE* DD genotype a higher level of systolic pressure compared to that in the *ACE* II and ID genotypes has been demonstrated [14].

**Table 1:** Distribution of *ACE* I/D and *MMP-7* A-181G genotypes in ESRD patients and controls

**Genotypes**	**Patients (%) **	**Controls (%)**	**OR**	**95% CI**	**P**
***ACE*** ** polymorphism** *					
II	13 (13.7)	18 (15.4)	1.0	-	-
ID	38 (40.0)	53 (45.3)	0.99	0.43-2.26	0.986
DD	44 (46.3)	46 (39.3)	1.32	0.58-3.02	0.504
					
***MMP-7*** ** Polymorphism** **					
AA	22 (22.2)	38 (33.3)	1.0	-	-
AG	71 (71.7)	60 (52.7)	2.04	1.09-3.82	0.026
GG	6 (6.1)	16 (14.0)	0.64	0.22-1.89	0.429

* Number of patients and controls were 95 and 117, respectively.

** Number of patients and controls were 99 and 114, respectively.

The frequency of *MMP-7* -181 AG genotype was significantly higher in ESRD patients (71.7%) than that in controls (52.7%, P=0.026) that was associated with 2.04 times ESRD risk (Table 1). There was no significant interaction between two variant alleles of *MMP-7* G and *ACE* D compared to both wild alleles of *MMP-7* A and *ACE* I. In kidney biopsy specimens from patients with lupus nephritis the expression of MMP-7 was correlated with chronicity index. This higher expression of MMPs could be a potential counter balance for increase in collagen expression and a physiological response to counter fibrotic injury [15]. Also, MMP7 might help to maintain tolerance through protective effects, because MMPs have been shown to contribute to tissue repair [16]. Briefly, the present study did not detect an association between the *ACE* I/D polymorphism and the risk of ESRD. However, the *ACE* D allele increased the risk of hypertension in ESRD patients. Also, the *MMP-7* AG genotype might be involved in the pathogenesis of ESRD.
